# Specific localization of nesprin-1-α2, the short isoform of nesprin-1 with a KASH domain, in developing, fetal and regenerating muscle, using a new monoclonal antibody

**DOI:** 10.1186/s12860-016-0105-9

**Published:** 2016-06-27

**Authors:** Ian Holt, Nguyen Thuy Duong, Qiuping Zhang, Le Thanh Lam, Caroline A. Sewry, Kamel Mamchaoui, Catherine M. Shanahan, Glenn E. Morris

**Affiliations:** Wolfson Centre for Inherited Neuromuscular Disease, RJAH Orthopaedic Hospital, Oswestry, SY10 7AG UK; Institute for Science and Technology in Medicine, Keele University, Keele, ST5 5BG UK; Institute of Genome Research (IGR), Vietnam Academy of Science and Technology (VAST), Hanoi, Vietnam; Cardiovascular Division, James Black Centre, King’s College, London, SE5 9NU UK; Dubowitz Neuromuscular Centre, Institute for Child Health and Great Ormond Street Hospital, London, WC1 1EH UK; Sorbonne Universités, UPMC Univ Paris 06, INSERM UMRS974, CNRS FRE3617, Center for Research in Myology, 47 Boulevard de l’hôpital, 75013 Paris, France

**Keywords:** *SYNE1*, Nuclear membrane, Monoclonal antibody, Cardiomyopathy, Emery-Dreifuss muscular dystrophy, Lamin A/C

## Abstract

**Background:**

Nesprin-1-giant (1008kD) is a protein of the outer nuclear membrane that links nuclei to the actin cytoskeleton via amino-terminal calponin homology domains. The short nesprin-1 isoform, nesprin-1-α2, is present only in skeletal and cardiac muscle and several pathogenic mutations occur within it, but the functions of this short isoform without calponin homology domains are unclear. The aim of this study was to determine mRNA levels and protein localization of nesprin-1-α2 at different stages of muscle development in order to shed light on its functions.

**Results:**

mRNA levels of all known nesprin-1 isoforms with a KASH domain were determined by quantitative PCR. The mRNA for the 111 kD muscle-specific short isoform, nesprin-1-α2, was not detected in pre-differentiation human myoblasts but was present at significant levels in multinucleate myotubes. We developed a monoclonal antibody against the unique amino-terminal sequence of nesprin-1-α2, enabling specific immunolocalization for the first time. Nesprin-1-α2 protein was undetectable in pre-differentiation myoblasts but appeared at the nuclear rim in post-mitotic, multinucleate myotubes and reached its highest levels in fetal muscle. In muscle from a Duchenne muscular dystrophy biopsy, nesprin-1-α2 protein was detected mainly in regenerating fibres expressing neonatal myosin. Nesprin-1-giant was present at all developmental stages, but was also highest in fetal and regenerating fibres. In fetal muscle, both isoforms were present in the cytoplasm, as well as at the nuclear rim. A pathogenic early stop codon (E7854X) in nesprin-1 caused reduced mRNA levels and loss of protein levels of both nesprin-1-giant and (unexpectedly) nesprin-1-α2, but did not affect myogenesis in vitro.

**Conclusions:**

Nesprin-1-α2 mRNA and protein expression is switched on during myogenesis, alongside other known markers of muscle differentiation. The results show that nesprin-1-α2 is dynamically controlled and may be involved in some process occurring during early myofibre formation, such as re-positioning of nuclei.

## Background

Nuclear envelope spectrin-repeat proteins (nesprins) form a structural link between the nuclear envelope and cytoskeletal filaments. The *SYNE1* gene, which encodes nesprin-1, was identified by yeast two-hybrid screening of fetal mouse post-synaptic membrane cDNA [1] and by differential screening of a rat vascular smooth muscle cell cDNA [2]. These early studies also identified a related gene, *SYNE2*, which encodes nesprin-2 [1, 2]. The protein products originally identified were, in fact, shorter C-terminal isoforms of larger proteins, nesprin-1-giant (1008kD) and nesprin-2-giant (792kD) [2–4]. The *SYNE1* gene on human chromosome 6q25 is also known as *MYNE1* [5] or *Enaptin* [6] and the *SYNE2* gene on human chromosome 14q23 is also known as *NUANCE* [3]. Both giant nesprins have calponin homology (CH) domains at their N-terminals that bind the actin cytoskeleton, and transmembrane Klarsicht-ANC-Syne-homology (KASH) domains at their C-terminals that bind to inner nuclear membrane SUN proteins in the luminal gap between inner and outer nuclear membranes [7, 8]. Additionally, on the nucleoplasmic side of the inner nuclear membrane, SUN proteins interact with A-type lamin components of the nuclear lamina. These linker of nucleoskeleton and cytoskeleton (LINC) complexes form a physical connection joining the cytoskeleton and the nucleus [9, 10]. Several human mutations in the C-terminal region of nesprin-1 are associated with Emery-Dreifuss muscular dystrophy (EDMD) and dilated cardiomyopathy [11–14]. Mutations in nesprin-interaction partners, emerin (*EMD*, [15] and lamin A/C (*LMNA*, [16]) account for about 50 % of EDMD cases (reviewed [17]).

*SYNE1* and *SYNE2* have multiple internal promoters which may give rise to shorter nesprin isoforms which are truncated at the N-terminus but have a common C-terminal region. Three additional members of the nesprin family (nesprin-3, nesprin-4 and KASH5) are similar to the shorter nesprin isoforms in that they lack N-terminal CH domains. Nesprin-3 (112kD) contains a plectin-binding domain at the N-terminal which interacts with intermediate filaments [18], nesprin-4 (44kD) interacts with microtubules via kif5b [19] and KASH5 (63kD) links to chromosomes via dynein [20, 21]. These functional products of 3 separate, but related, genes suggest possible related functions for the similar short products of the *SYNE1/2* genes.

We recently showed by qPCR that mRNAs for nesprin short forms were present at only very low levels in most of the 20 human tissues studied, but were significantly expressed in specific cell types or stages of development [22]. Thus, nesprin-1-α2 was found in skeletal and cardiac muscle only and nesprin-2-epsilon-1 was associated with cells at very early stages of development, while nesprin-2-epsilon-2 was expressed in cardiac, but not skeletal, muscle [22].

In the present study, we extended our qPCR studies of adult human tissues to different stages of skeletal muscle development and, finding that the mRNA for nesprin-1-α2 appeared only after myogenic differentiation, we developed a new monoclonal antibody specific for the unique N-terminal amino-acid sequence of nesprin-1-α2 for immunolocalization studies.

## Results

We have studied four stages of human muscle development: pre-differentiation, dividing myoblast cultures, differentiated myotube cultures expressing muscle-specific proteins, fetal muscle and adult muscle. We have also studied dystrophic muscle containing a significant proportion of immature fibres and muscle cultures with pathogenic mutations in nesprin-1 and lamin A/C.

### Nesprin-1-α2 mRNA appears during myogenesis in vitro and nesprin-1-giant mRNA increases

**Fig. 1:**
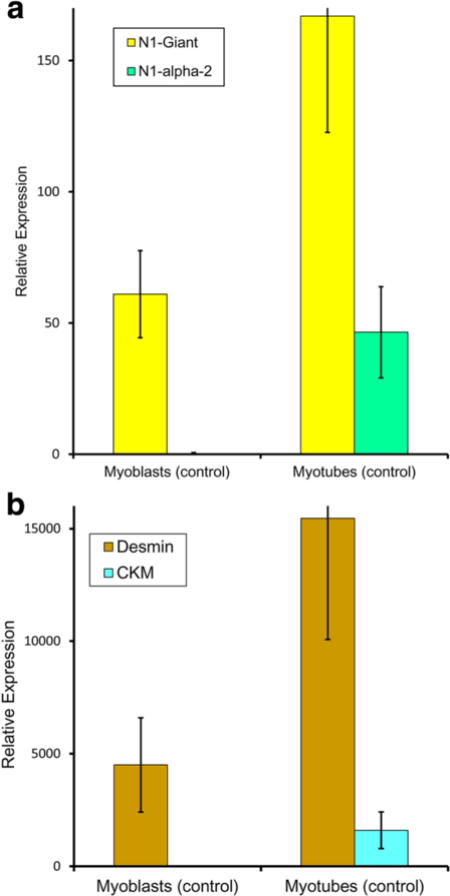
Expression of nesprin-1 isoforms and muscle markers in cultured control myoblasts and myotubes. Quantitative PCR to show mRNA expression relative to the expression of two endogenous controls. **a** nesprin-1-giant and nesprin-1-α2 and **b** desmin (intermediate filament) and muscle creatine kinase (differentiation marker). Bar charts represent the mean relative expression ± SEM of 5 control cell lines, with values for the individual cell lines shown in Table 1

mRNA levels in control human cells were determined by quantitative PCR (qPCR: Table 1) with appropriate standards and controls (see Methods). As expected, mRNA for the muscle differentiation marker, M-creatine kinase (CKM), was absent from dividing myoblasts, but increased markedly during muscle development (Fig. 1b). mRNA for the muscle cell marker protein, desmin, also increased; it was already present in the committed, though still dividing myogenic cells, as expected (Fig. 1b). Like CKM, nesprin-1-α2 mRNA was absent from myoblasts, but present in myotubes (Fig. 1a). Nesprin-1-giant mRNA was present in myoblasts, but also higher in myotubes (Fig. 1a). Other short isoforms of nesprin-1 did not make a major contribution to total nesprin mRNA content of cultured cells (Table 1). For these control myogenesis studies in vitro, five different transformed myoblast lines were used (Table 1), derived from control subjects of different ages and each individual cell line showed changes comparable to the mean values shown in Fig. 1a.

**Table 1:** Relative expression of mRNA of nesprin-1 isoforms and markers of differentiation in cultured human myoblasts and myotubes from control donors

	Control 5 days		Control 25 years		Control 41 years		Control 53 years		Control 79 years	
	Myoblast	Myotube	Myoblast	Myotube	Myoblast	Myotube	Myoblast	Myotube	Myoblast	Myotube
N1-Giant	61.1 ± 11.8 (5)	130.0 ± 12.8 (6)	88.3 ± 12.3 (6)	206.3 ± 17.3 (5)	47.7 ± 4.6 (4)	113.7 ± 19.3 (4)	47.4 ± 6.7 (5)	172.4 ± 22.1 (4)	60.5 ± 10.1 (4)	212.4 ± 12.1 (5)
	*P* < 0.001		*P* < 0.001		*P* < 0.001		*P* < 0.001		*P* < 0.001	
N1-β1	<1 (1)	<1 (1)	<1 (2)	<1 (2)	<1 (1)	<1 (1)	<1 (1)	<1 (1)	1.1 (1)	1.2 (1)
N1-β2	<1 (1)	1.2 (1)	<1 (3)	2.1 ± 0.8 (3)	<1 (1)	2.7 (1)	<1 (1)	<1 (1)	<1 (1)	1.2 (1)
N1-α1	nd (1)	nd (1)	nd (1)	nd (1)	nd (1)	nd (1)	nd (1)	nd (1)	nd (1)	nd (1)
N1-α2	<1 (5)	71.0 ± 9.5 (4)	<1 (3)	47.5 ± 9.3 (4)	<1 (5)	22.0 ± 1.3 (5)	<1 (5)	44.7 ± 9.1 (4)	<1 (3)	47.4 ± 4.3 (5)
	*P* < 0.001		*P* < 0.001		*P* < 0.001		*P* < 0.001		*P* < 0.001	
CKM	<1 (3)	1158 ± 116 (3)	<1 (3)	2847 ± 285 (3)	<1 (3)	740 ± 186 (3)	<1 (3)	1884 ± 104 (3)	2.1 ± 1.7 (3)	1362 ± 68 (3)
	*P* < 0.001		*P* < 0.001		*P* < 0.005		*P* < 0.001		*P* < 0.001	
Desmin	2773 ± 174 (3)	16434 ± 157 (3)	6931 ± 471 (3)	16966 ± 710 (3)	3784 ± 459 (3)	7103 ± 692 (3)	6532 ± 605 (3)	14799 ± 798 (3)	2484 ± 478 (3)	21973 ± 1803 (3)
	*P* < 0.001		*P* < 0.001		*P* < 0.005		*P* < 0.001		*P* < 0.001	

Results expressed as: Mean ± SD (n), “n” = number of repeat measurements. nd = Not detected

### Effects of pathogenic mutations in nesprin-1 and lamin A/C on mRNA levels of nesprin-1 isoforms during myogenesis

Myoblast cell lines were also derived from a muscular dystrophy patient with an early termination mutation in the nesprin-1 gene [23], located just before the start of the short nesprin-1-α2 isoform. One would expect all nesprin-1 mRNAs to be synthesised normally, though nesprin-1-giant mRNA might be less stable than normal, because of “nonsense-mediated decay”. In fact, consistent with reduced mRNA stability, the level of nesprin-1-giant mRNA was reduced by 78 % in both myoblasts and myotubes compared to the means of the 5 control cell lines and was lower than the corresponding values in each of the 5 controls individually (Table 2). Surprisingly, the mRNA for nesprin-1-α2 was also reduced by 85 % in the mutant myotubes, even though the mutation lies 5’ to its first exon. Levels of muscle marker mRNAs for CKM and desmin were unaffected by this mutation. Nesprins at the outer nuclear membrane are linked to lamin A/C in the nucleus via SUN proteins in the lumen of the nuclear membrane [10]. Therefore, we also studied myoblast cell lines with mutations in the *LMNA* gene, which encodes lamin A/C. Among the 3 myoblast lines from patients with 3 different mutations in the *LMNA* gene [24, 25], there was no consistent effect on the mRNA levels for nesprin-1 shown by all 3 *LMNA* mutant cell lines (Table 2). Nesprin-1-giant mRNA levels were very high in one *LMNA* mutant (L380S), but it is not clear, without the availability of additional similar cell lines, whether this is attributable to the specific mutation.

**Table 2:** Relative expression of mRNA of nesprin-1 isoforms and markers of differentiation in cultured human myoblasts and myotubes from patients with pathogenic mutations in Syne1 or LMNA

	SYNE-1 E7854X		LMNA L380S		LMNA del.K32		LMNA R249W	
	Myoblast	Myotube	Myoblast	Myotube	Myoblast	Myotube	Myoblast	Myotube
N1-Giant	13.9 ± 2.2 (6)	33.5 ± 5.0 (5)	102.1 ± 8.8 (5)	429.5 ± 42.4 (5)	48.3 ± 9.4 (4)	161.7 ± 33.6 (5)	53.4 ± 10.0 (4)	90.7 ± 19.0 (5)
	*P* < 0.001		*P* < 0.001		*P* < 0.001		*P* < 0.01	
N1-β1	<1 (2)	<1 (2)	1.0 (2)	<1 (2)	<1 (1)	<1 (1)	<1 (1)	<1 (1)
N1-β2	<1 (3)	1.8 ± 0.6 (4)	1.2 ± 0.1 (3)	3.0 (2)	<1 (1)	<1 (1)	<1 (1)	<1 (1)
N1-α1	nd (1)	nd (1)	nd (1)	nd (1)	nd (1)	nd (1)	nd (1)	nd (1)
N1-α2	<1 (3)	7.0 ± 4.1 (5)	<1 (4)	46.8 ± 6.2 (4)	<1 (3)	40.1 ± 7.8 (4)	<1 (4)	56.1 ± 11.2 (4)
	*P* < 0.01		*P* < 0.001		*P* < 0.001		*P* < 0.001	
CKM	3 ± 2 (3)	1290 ± 296 (3)	3 ± 1 (4)	1492 ± 206 (3)	<1 (4)	2346 ± 768 (3)	2 ± 1 (4)	1279 ± 320 (4)
	*P* < 0.005		*P* < 0.001		*P* < 0.005		*P* < 0.001	
Desmin	10331 ± 671 (3)	13038 ± 303 (3)	4064 ± 673 (6)	14334 ± 581 (4)	6084 ± 160 (4)	16701 ± 1321 (4)	7952 ± 441 (4)	11358 ± 231 (4)
	*P* < 0.005		*P* < 0.001		*P* < 0.001		*P* < 0.001	

Results expressed as: Mean ± SD (n), “n” = number of repeat measurements. nd = Not detected

### New monoclonal antibodies against nesprin-1 isoforms

**Fig. 2:**
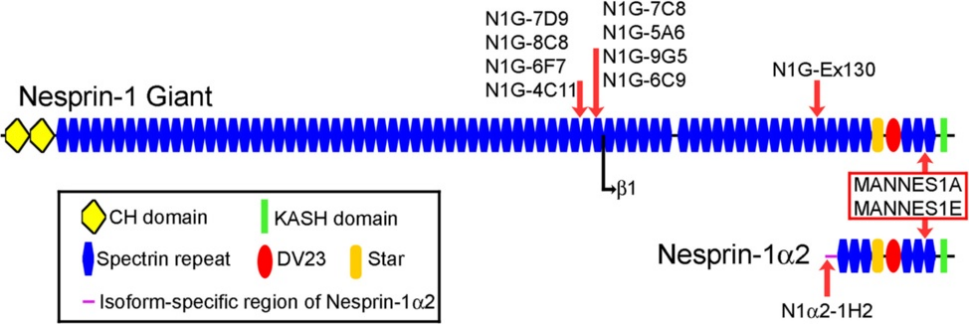
Pictorial representation of nesprin-1-giant and nesprin-1-α2 proteins with locations of monoclonal antibody (mAb) epitopes. Nesprin-1-α2 has a unique N-terminal sequence, but is otherwise identical to the C-terminal region of Nesprin-1-giant, including the unstructured and highly conserved “Star” region [28] and the alternatively spliced and highly conserved “DV23” exon [1, 4, 22, 28]. The N-terminal start point of nesprin-1-beta-1 relative to nesprin-1-giant, is shown as a black arrow. Red arrows indicate positions of the epitopes of mAbs against nesprin-1 that have been used in this study. Monoclonal antibodies MANNES1A and MANNES1E recognise the common C-terminal region of nesprin-1-giant and nesprin-1-α2 [26]. The new mAb, N1alpha2-1H2 recognises the unique N-terminal region of nesprin-1-α2 and the other new mAbs recognise nesprin-1-giant but not nesprin-1-α2

**Fig. 3:**
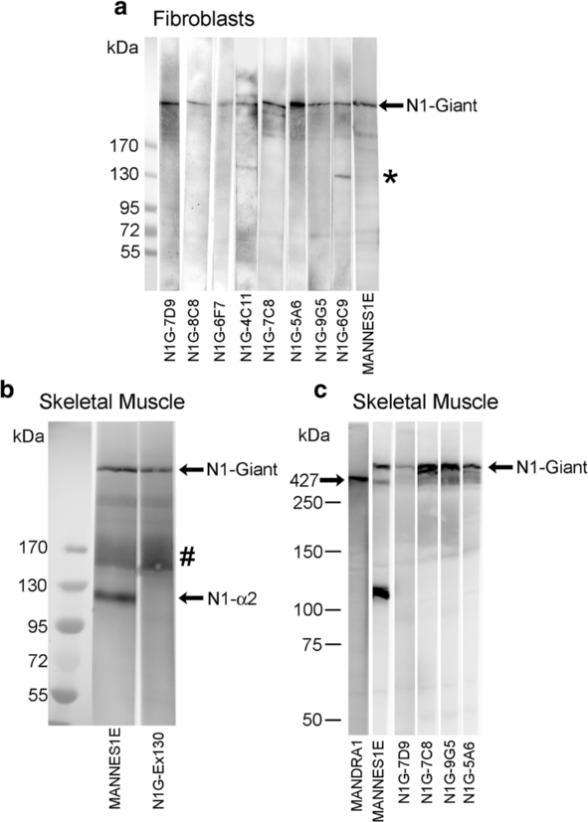
Western blots with nesprin-1 mAbs. Extracts of **a** cultured human dermal fibroblasts probed with the new mAbs against exons 81 to 86 of nesprin-1-giant and **b** normal adult human skeletal muscle probed with the new mAb N1G-Ex130 against exon 130 of nesprin-1-giant. The molecular weight marker used for blots (**a**) and (**b**) was: EZ-Run Pre-Stained Rec Protein Ladder (Fisher; Cat No: BP3603), upper molecular weight 170 kDa. Blot (**c**) is human skeletal muscle extract alongside Precision Plus Protein Standards (BioRad; Cat No: 161-0374) as molecular weight markers, upper molecular weight 250 kDa. Strips of blot (**c**) were probed with MANDRA1 to show dystrophin as a size marker (427 kDa). Bands with nesprin-1 mAbs were higher than the 427 kDa marker and consistent with nesprin-1-giant (1008 kDa). All blots include the C-terminal mAb against nesprin-1, MANNES1E [26]. * = likely non-specific bands; # = likely nesprin degradation products

Having established the changes in mRNA levels, it was necessary to determine whether protein levels changed in the same way. Since antibodies specific for short isoforms were not available, we produced new monoclonal antibodies and these are shown in Table 3. Human nesprin-1-α2 has a unique 31aa sequence at its amino-terminus and a synthetic peptide corresponding to amino acids 2-17 was linked to KLH via its C-terminal cysteine. This peptide conjugate was used to produce a monoclonal antibody (mAb: N1alpha2-1H2) specific for this short isoform, as described in “Experimental Procedures”. Another 16 amino acid peptide within exon 130 was used similarly to produce a second mAb, N1G-Ex130, which does not recognise nesprin-1-α2, but it does recognise nesprin-1-giant and would recognise beta isoforms. Beta isoforms are only expressed at low levels in skeletal muscle relative to the giant form (Table 3 and [22]), but to exclude them we also produced mAbs corresponding to exons 81-86 of the nesprin-1 gene (Table 3). These mAbs will recognise nesprin-1-giant but not the beta or alpha isoforms. The epitope locations of the new nesprin-1 mAbs, along with the MANNES1A and MANNES1E C-terminal mAbs (Randles et al., 2010 [26]), are illustrated in Fig. 2. All of the exon 81-86 mAbs recognised a band of nesprin-1-giant on western blots of a human skin fibroblast extract (Fig. 3a), the same band as recognised by a previously-established mAb, MANNES1E [26]. On western blot of human skeletal muscle extract, mAb N1G-Ex130, like MANNES1E, gave a band of nesprin-1-giant (Fig. 3b). For verification of the size of the nesprin-1-giant band, human skeletal muscle extract was run on a gradient gel along with a molecular weight marker with an upper band of 250 kDa (Fig. 3c). As an additional size marker, mAb MANDRA1 was used to show a band of dystrophin protein at 427 kDa. Nesprin-1 mAbs recognise bands that are larger than 427 kDa and are consistent with nesprin-1-giant at around 1008 kDa (Fig. 3c).

**Table 3:** Nesprin-1 monoclonal antibodies used in this study

mAb name	Isotype	Epitope	Western blot	IMF
		Unique sequence preceding Exon 131 of N1-Giant		
N1alpha2-1H2	IgG1	VVAEDLSALRMAEDGC (aa 2-17 of unique N1-alpha-2 sequence)	-	++ (myotubes, immature and regenerating fibres)
		Exon 130		
N1G-Ex130	IgG1	SKASEIEYKLGKVNDRC	+++	+++
		Exons 81-84		
N1G-7D9	IgG1	QDKLPGSSA (Exon 82)	+++	+
N1G-8C8	IgG1	-	++	+
N1G-6F7	IgG1	-	+	+
N1G-4C11	IgG2b	EMIDQLQDKLP (Exon 82)	+ (cross reaction)	+ (cross reaction)
		Exons 84-86		
N1G-7C8	IgG1	-	++	++
N1G-5A6	IgG1	-	++	+
N1G-9G5	IgG1	LGLYTILPSELSL (Exon 84)	+++	+
N1G-6C9	IgG1	LKIRDQIQDK (Exon 84-85)	+ (cross reaction)	+
		Exons143-146		
MANNES1A	IgG1	-	++	++++
MANNES1E [25]	IgG1	-	+++	+++

Except for MANNES1A and MANNES1E (Randles et al., 2010 [26]), all other Nesprin-1 mAbs in the table are reported here for the first time

Exon numbering is based on the SYNE1 transcript variant 1 mRNA (accession: NM_182961.3) and is the same as that used by Rajgor et al., 2012 [27]

**Fig. 4:**
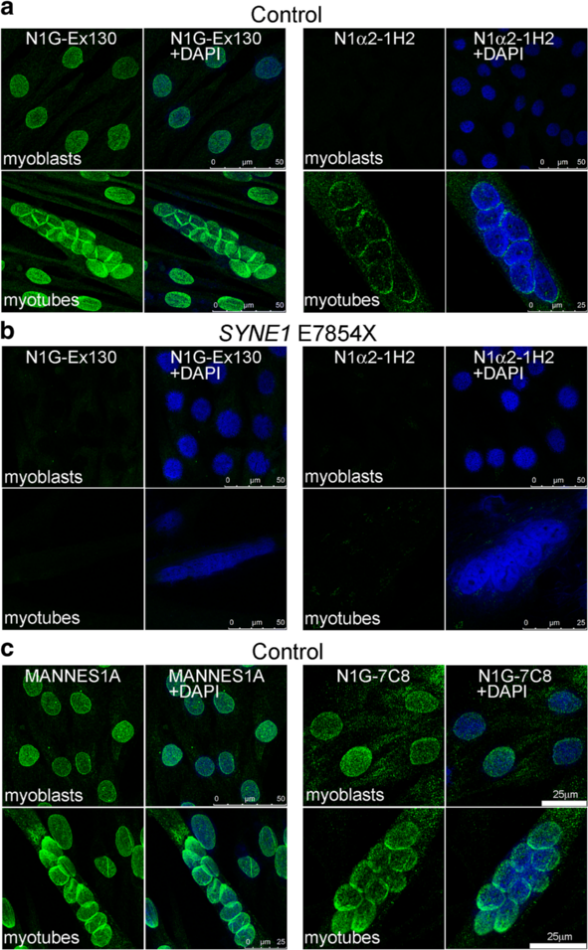
Immunofluorescent staining for nesprin-1 in cultured myoblasts and myotubes. Individual microscopic fields are shown as paired images. The left image of each pair shows nesprin-1 antibody stain (green) and the right image of each pair shows the merged antibody (green) and DAPI (blue) nuclear stain. **a** mAb N1G-Ex130 stained the nuclei of control myoblasts and myotubes whereas mAb N1alpha2-1H2 stained the nuclear envelope of control myotubes but not myoblasts. **b** The mAbs did not stain nesprin-1 mutant myoblasts or myotubes. **c** Similar to the image seen with N1G-Ex130, mAb MANNES1A and also the mAbs targeted against exons 81-86 (N1G-7C8 shown), stained the nuclear rim of control myoblasts and myotubes

The mAb N1G-Ex130 and the N1G-Exon 81-86 mAbs against the giant isoform recognise nesprin-1-giant, but would not be expected to recognise the short α2 isoform, on a western blot of adult human muscle, whereas MANNES1E does recognise a band of the size expected for nesprin-1-α2 (Fig. 3b and c). We cannot confirm that this band is nesprin-1-α2, because the α2-specific mAb, N1alpha2-1H2, does not work on western blots, but the specificity of the mAb is suggested by the absence of nuclear rim staining both in cells that do not express nesprin-1-α2, such as skin fibroblasts (not shown), and in myotubes with 85 % reduced α2 mRNA levels (*SYNE1* mutant cell line: Fig. 4b). The advantage of using N1G-Ex130 to detect nesprin-1-giant, instead of the exon 81-86mAbs, is that the exon 130 region does not encode any known N-terminal (KASH-less) isoforms of nesprin-1, whereas such isoforms containing the exon 81-86 region have been detected at the mRNA level [27]. There may be very few nesprin-1 sequences that are unique to the giant isoform.

### Nesprin-1-α2 protein is located mainly at the nuclear rim in myotube nuclei and is absent from myoblast nuclei

**Fig. 5:**
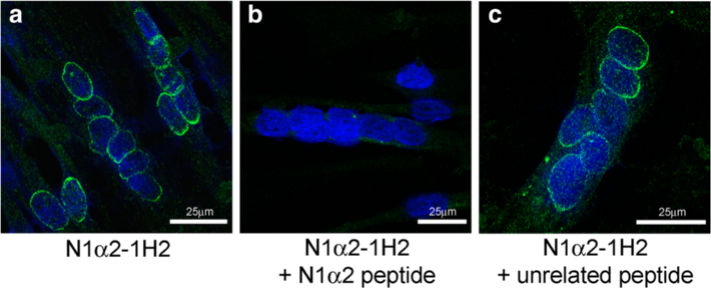
Peptide competition experiment. Localization of mAb N1alpha2-1H2 to the nuclear envelope of cultured myotubes (**a**) was neutralized when the mAb was blocked by pre-incubation with the immunizing peptide (**b**). Pre-incubation with an unrelated peptide did not prevent normal localization (**c**)

N1alpha2-1H2 mAb does not stain myoblasts, but does stain the nuclear rim in control myotubes (Fig. 4a), consistent with mRNA data showing the first appearance of nesprin-1-α2 mRNA at the myotube stage (Fig. 1). Pre-incubation of N1alpha2-1H2 with the peptide epitope used for immunization abolished immunostaining of myotubes, whereas control pre-incubation with an unrelated peptide did not (Fig. 5). In contrast to N1alpha2-1H2, MANNES1A (against both giant and alpha isoforms) stains the nuclear rim both in dividing myoblasts and in post-mitotic multinucleate myotubes (Fig. 4c). There is also some cytoplasmic staining in myotubes with N1alpha2-1H2 (Fig. 4a) and to a lesser extent, with MANNES1A (Fig. 4c). Nesprin-1-giant-specific mAbs at both exon 130 (N1G-Ex130, Fig. 4a) and exons 84-86 (N1G-7C8, Fig. 4c) confirm that nesprin-1-giant is present at the nuclear rim throughout myogenesis in vitro.

Myotube cultures of the early termination nesprin-1 mutant were negative for all nesprin-1 mAbs, including the N1alpha2-1H2 mAb (Fig. 4b). This may seem surprising since the stop codon is 5’ to the nesprin-1-α2 sequence, but qPCR shows that nesprin-1-α2 mRNA levels are only 15 % of control levels (Tables 1 and 2) and this may result in protein levels below the level of detection. The more surprising observation, therefore, is that the nesprin-1-α2 mRNA is so much reduced. There is the possibility of a truncated form of nesprin-1-giant protein, but qPCR (Tables 1 and 2) shows that this would probably exist at only 20-25 % of normal levels, even assuming it were as stable as the full-length protein. In fact, neither N1G-Ex130 (Fig. 4b) nor the nesprin-1-specific exon 81-86 mAbs (not shown) detected any nesprin-1-giant in the nesprin-1 mutant myotubes, confirming that truncated mutant protein was present at only very low levels, if at all.

### Nesprin-1-α2 protein is weakly expressed in mature muscle fibres, but is strongly expressed, alongside nesprin-1-giant in both fetal muscle and immature (regenerating) fibres of Duchenne muscular dystrophy muscle

**Fig. 6:**
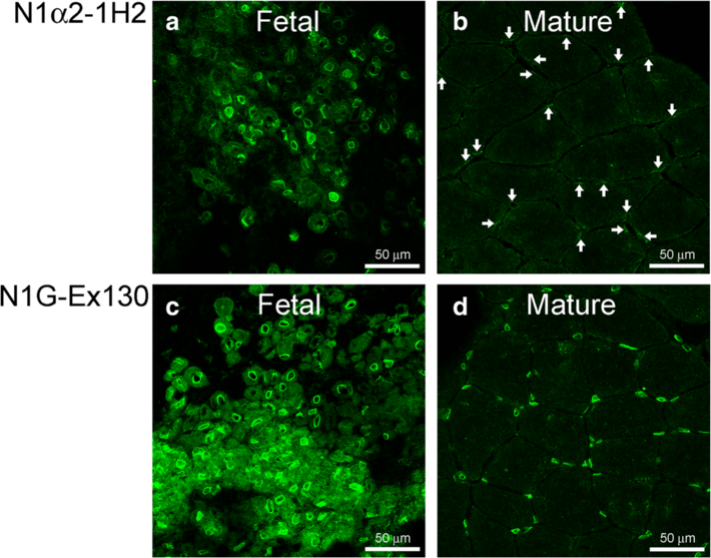
Immunofluorescent staining of fetal and mature human muscle for nesprin-1-α2 and nesprin-1-giant. **a** mAb N1alpha2-1H2 stained nuclei at the nuclear rim and gave less intense staining of cytoplasm in fetal muscle. **b** Nesprin-1-α2 was weakly stained in nuclei (white arrows) of mature muscle. Nesprin-1-α2 in mature muscle was much less obvious than that seen in fetal muscle. **c** Nesprin-1-giant (mAb N1G-Ex130) was found at the nuclear rim, with low level cytoplasmic stain, in fetal muscle. Nesprin-1-giant was also seen at the nuclear rim in adult skeletal muscle (**d**). Figure shows representative images of total of 3 fetal donors and 4 mature donors

**Fig. 7:**
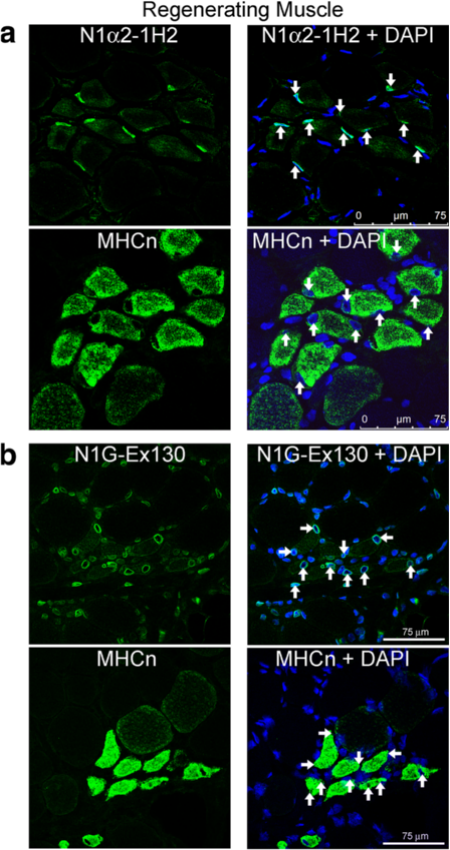
Immunofluorescent staining for nesprin-1 in human skeletal muscle sections with regenerating muscle fibres. Individual microscopic fields are shown as paired images. The left image of each pair shows antibody stain (green) and the right image of each pair shows the merged antibody (green) and DAPI (blue) nuclear stain. **a** Serial sections from a patient with Duchenne muscular dystrophy, stained with mAb N1alpha2-1H2 (upper frame) and neonatal myosin (MHCn; regenerating fibres; lower frame). Staining for nesprin-1-α2 was most intense at the nuclear rim, but also faintly in the cytoplasm, in those fibres that were regenerating. **b** Serial sections from the same donor, stained for nesprin-1-giant (mAb N1G-Ex130; upper frame) and neonatal myosin (MHCn; lower frame) indicated that nesprin-1-giant was detected in all nuclei, but staining for nesprin-1-giant at the nuclear rim was more intense, with faint cytoplasmic staining, in regenerating fibres. White arrows indicate the nuclei of regenerating fibres in the paired serial section

Nuclei of human fetal skeletal muscle were clearly labelled with the N1alpha2-1H2 mAb (Fig. 6a) and low level cytoplasmic staining was also seen with this mAb. The large central nuclei are typical of immature muscle fibres, whereas mature muscle nuclei are compressed and peripheral. We were surprised to find that the N1alpha2-1H2 mAb only gave a weak, low level stain in many mature muscle fibres (Fig. 6b), suggesting that the short isoform is either present at low levels and/or the mAb has reduced accessibility to the epitope. Rarely (<0.1 %), normal human adult muscle nuclei were observed that were strongly positive for nesprin-1-α2 (not shown). Nesprin-1-giant was found at the nuclear envelope of both fetal (Fig. 6c) and adult (Fig. 6d) skeletal muscle, with additional low level cytoplasmic stain in fetal muscle. Figure 6 shows representative images of N1alpha2-1H2 and N1G-Ex130 immunofluorescent staining of a total of 3 fetal donors and 4 adult donors. Furthermore, when we studied a muscle section from a Duchenne muscular dystrophy patient, we found that regenerating fibres (identified by staining serial sections with neonatal myosin antibody specific for immature fibres) had high levels of nesprin-1-α2 protein (Fig. 7a). Although mainly at the nuclear rim, N1alpha2-1H2 mAb also showed some staining of the cytoplasm of regenerating fibres, with mature muscle fibres being much weaker (Fig. 7a). Using N1G-Ex130 mAb to locate nesprin-1-giant, we found that this isoform was also higher in regenerating fibres, compared with mature fibres in the same section, and also showed faint cytoplasmic staining (Fig. 7b). Nesprin-1-giant was present at the nuclear rim of all nuclei in the muscle biopsy sections.

### Nesprin mRNA levels in fetal and adult human muscle

Both commercial and local sources were used for human fetal and adult skeletal muscle total RNAs, and total cDNAs were prepared by reverse transcription for qPCR quantification. Table 4 shows that nesprin isoform mRNA levels were highly variable between sources, although M-creatine kinase and desmin mRNA levels were significantly higher in adult, compared with fetal, muscle, as expected. Although Fetal 1 and Adult 1 (from the same commercial source) in Table 4 showed changes in nesprin mRNA consistent with the protein data (Figs. 4 and 6), they also expressed beta isoform mRNAs, not found in myotubes.

**Table 4:** Relative expression of mRNA of nesprin-1 isoforms and markers of differentiation in normal human skeletal muscle

	Fetal Skeletal Muscle			Adult Skeletal Muscle				
	Fetal 1	Fetal 2	Fetal 3	Adult 1 (30y)	Adult 2 (18y)	Adult 3 (67y)	Adult 4 (85y)	Adult 5
N1-Giant	269.0 ± 127.7 (8)	290.3 ± 36.6 (4)	5.3 ± 3.3 (5)	172.4 ± 27 (5)	113.6 ± 16.5 (4)	119.2 ± 30.4 (6)	47.5 ± 9.3 (5)	86.8 ± 25.9 (3)
N1-β1	2.4 ± 1.9 (5)	1 (2)	<1 (2)	21.3 ± 2.9 (4)	<1 (2)	<1 (2)	8.2 ± 3.0 (2)	9.8 ± 2.6 (3)
N1-β2	8.0 ± 1.9 (4)	124.4 ± 43.2 (8)	<1 (3)	4.9 ± 1.7 (4)	<1 (2)	1 (2)	<1 (3)	1 (2)
N1-α1	nd (1)	nd (1)	nd (1)	nd (1)	nd (1)	nd (1)	nd (1)	nd (1)
N1-α2	120.8 ± 33.9 (4)	66.2 ± 18.1 (5)	10.3 ± 2.3 (5)	17.8 ± 5.8 (4)	48.6 ± 12.7 (6)	140.3 ± 25.4 (5)	30.1 ± 5.3 (5)	55.4 ± 18.2 (3)
CKM	4237 ± 95 (4)	1611 ± 295 (4)	3404 ± 971 (5)	33117 ± 2320 (3)	89214 ± 10651 (4)	117928 ± 25917 (4)	20372 ± 1290 (4)	19033 ± 1851 (4)
Desmin	11928 ± 802 (3)	3260 ± 1034 (6)	14542 ± 3431 (5)	38904 ± 1567 (3)	84550 ± 20608 (4)	82022 ± 23108 (5)	37307 ± 617 (5)	27197 ± 4613 (4)

Results expressed as: Mean ± SD (n). “n” = number of repeat measurements. nd = Not detected

Human muscle tissue used for mRNA isolation will contain non-muscle cells, such as connective tissue, whereas the cultured cell lines are clonal and contain only desmin-positive myogenic cells. It is unclear, therefore, whether the beta isoform mRNAs in fetal and adult muscle are in myofibres (and therefore appear at some post-myotube stage) or in non-muscle components present in variable proportions in different muscle samples used for RNA extraction; this question can only be resolved with antibodies specific for the beta isoforms and these are not yet available. When we expanded our sampling to three fetal and five adult RNA preparations (Table 4), the results for all the nesprin-1 isoforms were much less consistent. It is possible that nesprin-1 mRNA content is variably affected by tissue storage and methods of RNA extraction, since the qPCRs themselves were quite reproducible and gave good dissociation curves. Nesprin-1-α2 mRNA is barely detectable in dividing myoblasts, but accounts for up to one-third of all the nesprin-1 mRNA in adult muscle (Table 4), a proportion consistent with the western blot in Fig. 3b and c.

## Discussion

N1alpha2-1H2 is the first anti-nesprin-1 antibody that is specific for a single isoform since it recognises a sequence encoded by the unique first exon of nesprin-1-α2. Antibodies against the N-terminal region of nesprin-1-giant will not recognize any other KASH+ isoforms but a number of shorter N-terminal isoforms are now known [6, 28], including CPG2 (109kD) [29], GSRP-56 (56 kD) [30] and Drop1 [31]. More recently, Rajgor et al, [10, 27] have shown numerous isoforms that cover much of the N-terminal half of the molecule, although their abundance and tissue distribution has not been precisely established. For this reason, we have extended the repertoire of available antibodies by producing a new mAb, N1G-Ex130, which recognises only giant and beta isoforms of nesprin-1, plus a panel of new mAbs that recognises nesprin-1-giant and NOT beta isoforms (though they would also recognize some N-terminal KASH-less isoforms, if they were present).

In addition to N-terminal isoforms lacking large C-terminal regions, there are also known isoforms that lose only a short KASH sequence by alternative splicing (e.g. the last 99 amino-acids; [32]) and are otherwise similar to the known KASH+ isoforms. There are currently no antibodies that distinguish these KASH-less forms from their KASH+ equivalents. Such KASH-less forms may have a putative nuclear localization signal (over 800 amino-acids from the nesprin-1 C-terminus [2]) and the KASH-less form of nesprin-1-giant in cerebellum has been shown to locate to the cytoplasm [32]. In embryonic stem cells, however, KASH-less forms of the related protein, nesprin-2, located to the nucleoplasm [22].

Using the new mAb tools described here for the first time, we have shown that nesprin-1-α2, the principal short form of nesprin-1 in cardiac and skeletal muscle [1, 22], appears after myoblast differentiation into early myotubes, remains at high levels in immature muscle fibres and becomes very weakly present by immunolocalization in most mature, adult muscle fibres (Figs. 6 & 7). Nesprin-1-giant, the main product of the *SYNE1* gene in most, if not all, tissues, was present at all stages of muscle development (Figs. 4 and 6), though its levels were also somewhat elevated in immature fibres relative to mature fibres (Fig. 7b). We have previously reported a decrease in nesprin-1 and an increase in nesprin-2 during the transition from immature to mature muscle fibres [26].

The clonal myoblast cell lines used in this study are a valuable resource because, unlike primary myoblast cultures, they do not contain variable proportions of non-muscle cells, such as fibroblasts. Immortalization does not seem to have affected their differentiation potential, we did not observe any effect of donor age on nesprin mRNA expression (Table 1) and the mRNA data from qPCR were consistent with the protein data from antibody studies (Fig. 4).

Fetal and adult muscle RNA samples, however, whether obtained commercially as extracted total RNA or locally as tissue stored for RNA extraction, gave very variable results in qPCR (Table 4). It seems likely that variability between samples, both in their “non-muscle” tissue content and in the storage and extraction conditions, might affect the levels of some mRNA species. Indeed, Table 4 suggests that low abundance mRNAs, like those for nesprins, give less consistent qPCR results than abundant species, like creatine kinase and desmin mRNAs. However, the mRNA for nesprin-1-α2 was detected by qPCR in adult muscle (Table 4) in amounts, relative to nesprin-1-giant, consistent with the 111kD band in western blot (Fig. 3b and c). In other words, the mRNA was certainly present in adult muscle, although nesprin-1-α2 protein was detected weakly by immunofluorescence microscopy in normal adult muscle. One possibility is that the epitope recognized by our mAb at the N-terminus of nesprin-1-α2 becomes masked in mature fibres in situ; it is not possible to test this hypothesis using different nesprin-1-α2-specific mAbs because the unique N-terminal immunogen sequence for this isoform is very short. This seems the simplest way to reconcile qPCR, western blot and immunofluorescence data at the present time.

A less likely possibility is that not all of the western blot band at around 111kD detected by mAb MANNES1E (Fig. 3b and c) is actually nesprin-1-α2; in a previous study, we showed that some prominent protein bands on western blots of skeletal muscle extracts, previously interpreted as known isoforms, were likely degradation products of the giant isoforms, since the corresponding mRNAs were barely detectable by qPCR [22]. It is also possible, but very unlikely, that larger muscle samples used for RNA extraction contain nesprin-1-α2-positive cells that are not seen in our small biopsy samples for immunofluorescence microscopy.

Missense mutations in the 3’-region of *SYNE1* usually result in autosomal-dominant Emery-Dreifuss muscular dystrophy or inherited cardiomyopathy, with nesprin-1 function impaired, though perhaps not abolished [11–14]. The homozygous *SYNE1* early termination mutation produces a severe, congenital muscular dystrophy phenotype, affecting several tissues [23], probably because expression of KASH+ nesprin-1 is massively reduced. If the expression of nesprin-1-α2 had been unaffected, as predicted from the position of the early stop codon, it might have thrown light on roles for nesprin-1-giant that cannot be replaced by the short isoform, nesprin-1-α2. However, both the mRNA and protein levels of nesprin-1-α2 were as much affected as was nesprin-1-giant by the mutation, although there does not appear to be any simple explanation for the reduced levels of nesprin-1-α2 mRNA. It is interesting that the mutation did not seem to affect in vitro myogenesis, measured by either myotube formation (Fig. 4b) or creatine kinase mRNA expression (Table 2). Nuclear re-positioning to the fibre periphery, however, does not occur until later stages of muscle development.

The detection of cytoplasmic nesprin-1 isoforms (both giant and alpha-2) in fetal muscle fibres, and to a lesser extent in myotubes, in addition to their principal location at the nuclear membrane, is not altogether surprising, since non-nuclear rim localizations in some circumstances have been reported previously [22, 26, 33]. The possibility that these are KASH-less splice variants, unable to attach to the nuclear membrane, cannot be excluded.

Although we have no evidence that nesprin-1-α2 is involved in movement of nuclei during muscle formation, there is circumstantial evidence to suggest that this is one possibility. Nuclear positioning is disrupted in nesprin-1 knockout mice [34, 35] and nesprin-1-giant is known to link the nuclear rim to the actin cytoskeleton [2, 36]. Although nesprin-1-α2 is unable to bind to actin via CH domains, it does share with the giant form, in its highly-conserved STAR domain [28], a LEWD motif that appears to be involved in binding kinesins [37]. Kinesins act as a linker to the microtubule system, which is involved in active movement of nuclei [38], including the movement of myonuclei along the longitudinal axis of the developing myotube [39–41]. This LEWD motif is the only part of the STAR domain shared by nesprin-4, a 44kD KASH-domain protein also known to bind microtubules via kinesins, but with a distribution that may be limited to tissues with secretory epithelial cells [19]. KASH5 lacks a LEWD motif and appears to bind to microtubules via dynein; like nesprin-4, it also exhibits a limited range of expression in spermatocytes, bone marrow and fetal liver [21]. It is possible that KASH proteins in the size range of 44-111kD, such as nesprin-1-α2, carry out similar functions related to nuclear movements, but in specific tissues or at specific stages of development.

## Conclusions

Expression of mRNA and protein of nesprin-1-α2, the skeletal and cardiac muscle-specific isoform of nesprin-1, is switched on during myogenesis, mirroring the expression of muscle-creatine kinase. The dynamic profile of nesprin-1-α2 expression suggests that it may be involved in some process occurring during early myofibre formation, such as re-positioning of nuclei. If the weak staining of nesprin-1-α2 in mature fibres is due to epitope masking, we might speculate that nesprin-1-α2 is carrying out one function in immature fibres, when it is accessible to antibody, and a different function in mature fibres, when antibody access is restricted.

## Methods

### Muscle cell culture

Clonal immortalized myoblast cell lines (Table 5) were from five human control donors without neuromuscular disease (aged 5 days and 25, 41, 53 and 79 years), from a congenital muscular dystrophy patient with a homozygous premature stop mutation in the nesprin-1 gene (Nucleotide: 23560 gaa to taa; Protein: E7854X; [23] and from three congenital muscular dystrophy patients with mutations in the lamin A/C gene (Protein: L380S, del.K32 and R249W; [24, 25]. They were immortalized by transduction with human telomerase reverse transcriptase (hTERT) and cyclin-dependent kinase-4 (Cdk4) containing retroviral vectors, at the Institut de Myologie, Paris, as described previously [42]. They were cultured in skeletal muscle cell growth medium (Cat No: C-23060; PromoCell GmbH, Heidelberg, Germany) containing supplement mix (Cat No: C-39365; PromoCell) with 20 % Fetal Bovine Serum (Cat No: 10270; Gibco; ThermoFisher Scientific, Paisley, UK). Differentiation was induced at 80 % confluency by washing the adherent myoblasts in medium lacking serum and then culturing in DMEM (Cat No: 31966-021; Gibco; ThermoFisher Scientific) supplemented with Insulin (1721nM), Transferrin (68.7nM), Selenium (38.7nM) (ITS-X; Cat No: 51500-056; Gibco; ThermoFisher Scientific) and Penicillin-Streptomycin (Cat No: DE17-603E; Lonza; Verviers, Belgium). After a further 4 days of cell culture, over 80 % of the cells had fused into myotubes.

**Table 5:** Immortalized human myoblasts

Name	Donor	Donor Muscle
C5d	Newborn (5.5 day), female	Quadriceps
C25yr	25 years, male	Semitendinosus
C41yr	41 years, male	Pectoralis Major
C53yr	53 years, male	Quadriceps
C79yr	79 years, female	Quadriceps
*Syne-1* E7854X	16 years. *SYNE1* c.23560 G > T, p.Glu7854* Homozygous	Paravertebral
*LMNA* L380S	12 years, male. *LMNA* c.1139T > C, p.Leu380Ser Heterozygous	Paravertebral
*LMNA* del.K32	5 years, female. *LMNA* c.94_69delAAG, p.Lys32del Heterozygous	Gastrocnemius
*LMNA* R249W	3 years, male. *LMNA* c.745C > T, p.Arg249Trp Heterozygous	Deltoid

Control, Syne-1 mutant [23] and LMNA mutant myoblasts [24, 25], immortalised as described [42]

### RT-PCR and qPCR

Total RNA was prepared from cultured cells and from skeletal muscle samples using RNeasy Plus Mini Kit (Qiagen) and quantified with a NanoDrop ND-1000 spectrophotometer. Human skeletal muscle total RNA were obtained from the following sources: Fetal 1 (19 week female fetus; cat no: T5595-7587; lot no: L14020669) and Adult 1 (30 year female; cat no: T5595-7379; lot no: L11033012) were both purchased from United States Biological (Swampscott, MA 01907). Fetal 3 (18 week female fetus; cat no: 540181; lot no: 0006260887) and Adult 4 (85 year female; cat no: 540029; lot no: 0006167155) were both purchased from Agilent Technologies (Stockport, Cheshire, SK8 3GR, UK). Adult 5 from the First Choice Human Total RNA Survey Panel (cat no: AM6000; lot no: 1004067; [22]) was purchased from Ambion Inc (Austin, TX 78744). Fetal 2 muscle was obtained from the MRC Centre for Neuromuscular Disease Biobank, London. Adult 2 muscle (18 year male) and Adult 3 muscle (67 year female) were obtained during routine surgery at RJAH Orthopaedic Hospital, Oswestry. Total RNA (maximum of 2.5μg in a 20μL reaction) was reverse transcribed (SuperScript VILO cDNA Synthesis Kit; Applied Biosystems) and then diluted with sterile water, in order to achieve the cDNA equivalent of 10ng total RNA for each 20μL reaction in the qPCR plate.

Forward primers for the short isoforms of nesprin-1 were designed to recognise unique sequences in the 5’ UTR of each isoform [22]. Primer sequences for M-creatine kinase (accession: NM_001824) were F: GCTCGTCCGAAGTAGAACAGGTG and R: GGTTGGAACTCTGGTTGAAACTG (282 bp product size) and for desmin (accession: NM_001927) were F: GCTCAACGTGAAGATGGCCCT and R: CTGCTGCTGTGTGGCCTCACT (223 bp product size). Primer pairs were tested by conventional PCR and products were confirmed by DNA sequencing [22]. Primer pairs were chosen which gave dissociation curves with single peaks.

Relative quantitative PCR was performed with an ABI 7500 Real Time PCR system (Applied Biosystems) using SYBR green detection, as previously described [22]. For each preparation of cDNA, two endogenous controls (Beta-actin and GAPDH) were amplified along with each target sequence. The 2^-∆CT^ method [43, 44] was used to calculate the quantity of target transcripts relative to the two endogenous reference transcripts. The C_T_ (cycle threshold) value was plotted against the log cDNA dilution in order to calculate the efficiency of the primer pairs [44].

### Hybridoma production

#### Peptide immunogens

The peptide VVAEDLSALRMAEDGC, with Keyhole Limpet Hemocyanin (KLH) conjugated to its C-terminus (Davids Biotechnologie GmbH, Regensburg, Germany), was used to immunize CD1 mice for production of mAbs against the unique N-terminal sequence of Nesprin-1-α2. The peptide SKASEIEYKLGKVNDRC, with KLH conjugated to its C-terminus (AltaBioscience, Birmingham UK), was used to immunize Balb/C mice for production of mAbs against a sequence within exon 130 of Nesprin-1-giant. For hybridoma production (described in detail previously: Nguyen thi Man and Morris, 2010 [45]), spleen cells from immunized mice were fused with mouse myeloma cell line Sp2/O and 960 wells were screened by ELISA against unconjugated peptides. ELISA-positives were screened by immunofluorescence microscopy on unfixed human muscle sections and western blotting with human skeletal muscle extracts (for exon 130 mAbs), before cloning by limiting dilution.

#### Recombinant fusion protein immunogen

Nesprin-1-giant cDNA (corresponding to amino acids 5209-5570) in pGEX vector was used to transform *E. coli* BL21(DE3) which were induced by IPTG to give a GST-fusion protein. After incubation the cells were washed with TNE buffer and sonicated sequentially with TNE, 2M urea, 4M urea, 6M urea and 8M urea. Fusion protein in the 6M urea fraction was used in the protocol for immunization of Balb/C mice for production of mAbs [45]. ELISA plates were coated with either the GST-nesprin-1 amino acids 5209-5570 fusion protein used for immunization or with an unrelated GST-fusion protein (GST-MSH3), in order to eliminate those mAbs reacting with the GST fusion tag. For further refinement of the epitope positions, ELISAs were also performed with plates coated with recombinant protein GST-nesprin-1-giant amino acids 5377-5570 (nesprin-1 isoform p23, Accession No: JQ754364), in order to identify those mAbs with epitopes at the C-terminal end of the original immunogen. ELISA-positives were screened by immunofluorescence microscopy and western blotting, before cloning by limiting dilution. Further epitope mapping was performed by testing the mAbs for immunolocalization with COS-7 cells transfected with pCMV/nesprin-1-beta-1 (amino acids 1-97). These mAbs were therefore classed as reacting with nesprin-1-giant exons 81-84 (amino acids 5209-5376) or nesprin-1-giant exons 84-86 (amino acids 5377-5476), which does not include any sequence from nesprin-1-beta-1.

Other mAbs used in this study were: MANNES1A (for immunofluorescence microscopy) and MANNES1E (for western blot), both of which recognise the C-terminal region of nesprin-1 [26].

### SDS-polyacrylamide gel electrophoresis and Western blotting

Cell lysis buffer (125 mM Tris pH 6.8, 2 % SDS, 5 % 2-beta mercaptoethanol, 5 % glycerol with protease inhibitors: Sigma P8340 and 1mM PMSF) was used for the extraction of cultured cells and tissue lysis buffer (50mM Tris pH 6.8, 1 % EDTA, 10 % SDS, 5 % beta mercaptoethanol, 10 % glycerol with protease inhibitors) was used for the extraction of tissue samples (250 mg/ml). Bromophenol blue was added to the samples which were then boiled and separated by SDS-PAGE using 4 to 12 % polyacrylamide gels (Ref: NW04125BOX; ThermoFisher Scientific) and then electroblotted onto nitrocellulose membranes (Protan BA85, Whatman). Non-specific sites were blocked with 5 % skimmed milk protein and the membranes then incubated with monoclonal antibody supernatants (diluted: 1/10, except MANNES1E: 1/50 and MANDRA1: 1/100), followed by washing and incubation with secondary antibody (peroxidase-labelled rabbit anti-mouse immunoglobulins; 1/1000, Dako, Denmark). Antibody reacting bands were detected with SuperSignal West Femto chemiluminescent reagent (Cat No: 34094; ThermoFisher Scientific) and visualized with a ChemiDoc Touch imaging system (BioRad Ltd.).

### Immunofluorescence microscopy

Immunohistochemistry was performed on cultured cells on coverslips that had been fixed with 50 % acetone, 50 % methanol and then washed with PBS and also performed on unfixed cryostat sections of human muscle. Monoclonal antibodies in culture supernatants were diluted and incubated on specimens for 1 h. N1alpha2-1H2 was diluted 1:1 to 1:3 in PBS and all other nesprin-1 mAbs were diluted 1:3 in PBS. Following incubation with the monoclonal antibody, specimens were washed with PBS and then incubated with goat anti-mouse ALEXA 488 (Molecular Probes, Eugene, Oregon, USA) secondary antibody (diluted to 5μg/ml in PBS containing 1 % horse serum, 1 % fetal bovine serum, 0.1 % BSA). After 50 min, DAPI (diamidino phenylindole at 200 ng/ml) was also added to specimens to counterstain nuclei. After incubation for 10 min with DAPI, specimens were washed with PBS and mounted in Hydromount (Merck). Images were acquired by sequential scanning with a Leica TCS SP5 spectral confocal microscope (Leica Microsystems, Milton Keynes, UK). For peptide competition experiments, mAb N1alpha2-1H2 was pre-incubated with 1mg/ml unconjugated peptide (VVAEDLSALRMAEDGC) or unrelated control peptide (IFSHQQVKKLKETFAFIQQLC) for 1 h at room temperature.

## Abbreviations

CH, Calponin homology; CKM, Creatine kinase, muscle; EDMD, Emery-Dreifuss muscular dystrophy; KASH, Klarsicht, ANC-1 and Syne homology; KLH, Keyhole limpet hemocyanin; LINC, Linker of Nucleoskeleton and Cytoskeleton; SUN, Sad-1, UNC-84
